# The effect of the Smart Health Continuous Feedback For Elderly Exercise (SHe CoFFEE) program on mobility: a randomized controlled pilot study

**DOI:** 10.3389/fpubh.2024.1442064

**Published:** 2024-09-13

**Authors:** Jungeun Yi, Sunhee Lee, Seon Heui Lee

**Affiliations:** ^1^College of Nursing, The Catholic University of Korea, Seoul, Republic of Korea; ^2^College of Nursing, Gachon University, Incheon, Republic of Korea

**Keywords:** exercise, information and communication technology, older adults, smart healthcare, mobility

## Abstract

**Objectives:**

This study determined the effectiveness of a comprehensive home-based online exercise program called “Smart Health Continuous Feedback for elderly exercise (SHe CoFFEE)” on mobility.

**Methods:**

Sixty community-dwelling seniors were randomized in a 1:1 ratio to intervention or control group. Intervention was an 8-week “SHe CoFFEE” program, featuring continuous feedback, self-monitoring, and fall prevention exercises. The primary outcome was mobility, measured at 8 weeks with the Timed Up and Go (TUG) test. Secondary outcomes included TUG at 4 weeks, 10 m walking test, 30-s chair stand test, falls efficacy scale, activity-specific balance confidence, and Euro Quality of Life 5 Dimensions 5 Levels.

**Results:**

At 8 weeks, the intervention group showed improved TUG scores compared with the control group (MD = −1.87, 95% CI, −2.60 to −1.14; *η_p_*^2^ = 0.326) in the adjusted intention-to-treat analysis. The per-protocol analysis data showed similar results. All secondary outcomes apart from quality of life improved with intervention to a greater degree than in control.

**Conclusion:**

Smart healthcare and self-managed exercise programs may be viable for community-based health promotion and fall prevention in older adults unfamiliar with online technology.

## Introduction

Both in terms of absolute numbers and as a percentage of the population, the global senior population is increasing and the percentage of people in the world who are 65 or older is expected to rise from 10% in 2022 to 16% in 2050 (1). Korea is expected to become a super-aged society in 2025 with 20.3% of the population aged 65 and older compared to 16.3% in 2021, making Korea the fastest-aging among OECD countries (2). For people over 65, falls are the primary cause of injury-related deaths, and the age-adjusted rate of fall-related deaths is rising (3). Falls are the primary concern for older adults transitioning from independent living to institutional care (4) and are a major threat to their quality of life, often leading to a decline in their ability for self-care and participation in physical and social activities (5, 6).

Physical activity in older adults helps prevent falls and fall-related injuries as well as maintain bone health and prevent functional deterioration (7). To increase physical activity, multifactorial interventions for older adults have been investigated. Lee and Yu verified the effects of the intervention including exercise, education, environmental modification, and mobility aids (8). Brickwood et al. developed the intervention using continuous feedback (9) and Steinert et al. used self-assessment to motivate older adults (10). These programs have been more effective in improving exercise adherence (8–10). Additionally, multifactorial interventions aiming to enhance older individuals’ physical function and knowledge, thereby improving their safety (11), have been shown to reduce falls by approximately 25% (5). In one study, for example, group exercise improved balance and muscle strength irrespective of fall risk (12).

However, multifactorial interventions in offline settings may be costly and the effectiveness may vary due to factors like location, weather, and travel distance. Moreover, the coronavirus pandemic has resulted in increased avoidance of gatherings for group exercise. In the United States, for example, the onset of COVID-19 was associated with a significant decrease in mobility (13). Ever since the pandemic, the amount of daily physical activity that individuals were engaged in has decreased significantly around the globe (14). In a Korean study, about half of adults 19 and older reported reduced physical activity during the pandemic (15). Therefore, there is a need for online interventions using smart healthcare, that is health service systems that use technology such as wearable devices and mobile internet (16). Regarding smart healthcare exercise interventions, although this does not apply to all older adults, many are unfamiliar with internet devices and tend to prefer group exercises over individual ones (17). A study by van der Bij and associates for example demonstrated greater participation of older adults in group exercises as compared to solo exercise programs (18), indicating that social interaction should be taken into account when implementing exercise plans for older people. Consequently, we designed a smart healthcare intervention enabling both-way conversations during group exercise sessions. Encouraging older adults to consistently participate in online exercise programs can be challenging. Some intervention programs have used exercise videos (19) or game programs (20) to allow older adults to exercise independently. Older adults often find smart healthcare interventions challenging, underscoring the importance of tailoring interventions to their needs. Hence, our objective was to increase engagement in smart healthcare interventions among older individuals. To achieve this, we developed a program with continuous feedback, self-assessment, and real-time group, and individual exercises through a user-friendly mobile application. The present study aims to ascertain the superiority of an 8-week structured, comprehensive home-based exercise program called “Smart Health Continuous Feedback for elderly exercise (SHe CoFFEE)” over control concerning the mobility of community-dwelling older adults in South Korea.

### Ethical approval

The study was approved by the Institutional Review Board of G University (1044396-202203-HR-067-01) in April 2022 and was conducted following the tenets of the Declaration of Helsinki.

## Methods

### Study design

This was a randomized, controlled pilot study with a parallel-group design and assessor-blinding. As shown in Table 1, the “SHe CoFFEE” program was implemented at two different time points. The first program began for the experimental group immediately after baseline measurements. The second program started for the control group after the final assessment of this RCT at 8 weeks. Measurements were taken at three-time points: before the intervention, 4 weeks after it started, and at the end of the intervention. The study design was developed with reference to previous studies (21, 22) conducted over 8 weeks with older adults in the community. Participants visited the public health center every 4 weeks for outcome assessments. This randomized controlled study followed the Consolidated Standards of Reporting Trials (CONSORT) recommendations (23) and was registered on the cris.org website (KCT0007288).

**Table 1:** Study design for SHe CoFFEE program.

Experimental group	Baseline measurement	1st SHe CoFFEE program			
		Program contents	4-week follow-up measurement	8-week follow-up measurement	
	General Assessment TUG 10MWT 30CST Fear of falling balance confidence quality of life	Continuous feedback Self-monitoring Fall prevention exercise	TUG 10MWT 30CST Fear of falling balance confidence quality of life	TUG 10MWT 30CST Fear of falling balance confidence quality of life	
Control group	Baseline measurement		4-week follow-up measurement	8-week follow-up measurement	2nd SHe CoFFEE program
	General Assessment TUG 10MWT 30CST Fear of falling balance confidence quality of life		TUG 10MWT 30CST Fear of falling balance confidence quality of life	TUG 10MWT 30CST Fear of falling balance confidence quality of life	Continuous feedback Self-monitoring Fall prevention exercise

### Participants

The participants were generally healthy older adults residing in the community, recruited from the N Public Health Center in Incheon, South Korea. They were registered as vulnerable populations at the health center, which had a higher proportion of women. In South Korea, women’s participation rates in social engagement tend to be higher than men’s (24). Since the measurement tools for evaluating the outcomes of the mobility exercise program required participants to walk independently, only those who could walk were included in the study. Community-visiting nurses at the Public Health Center facilitated the recruitment process. A total of 93 participants were recruited in June 2022, and informed consent was obtained at that time.

**Figure 1:**
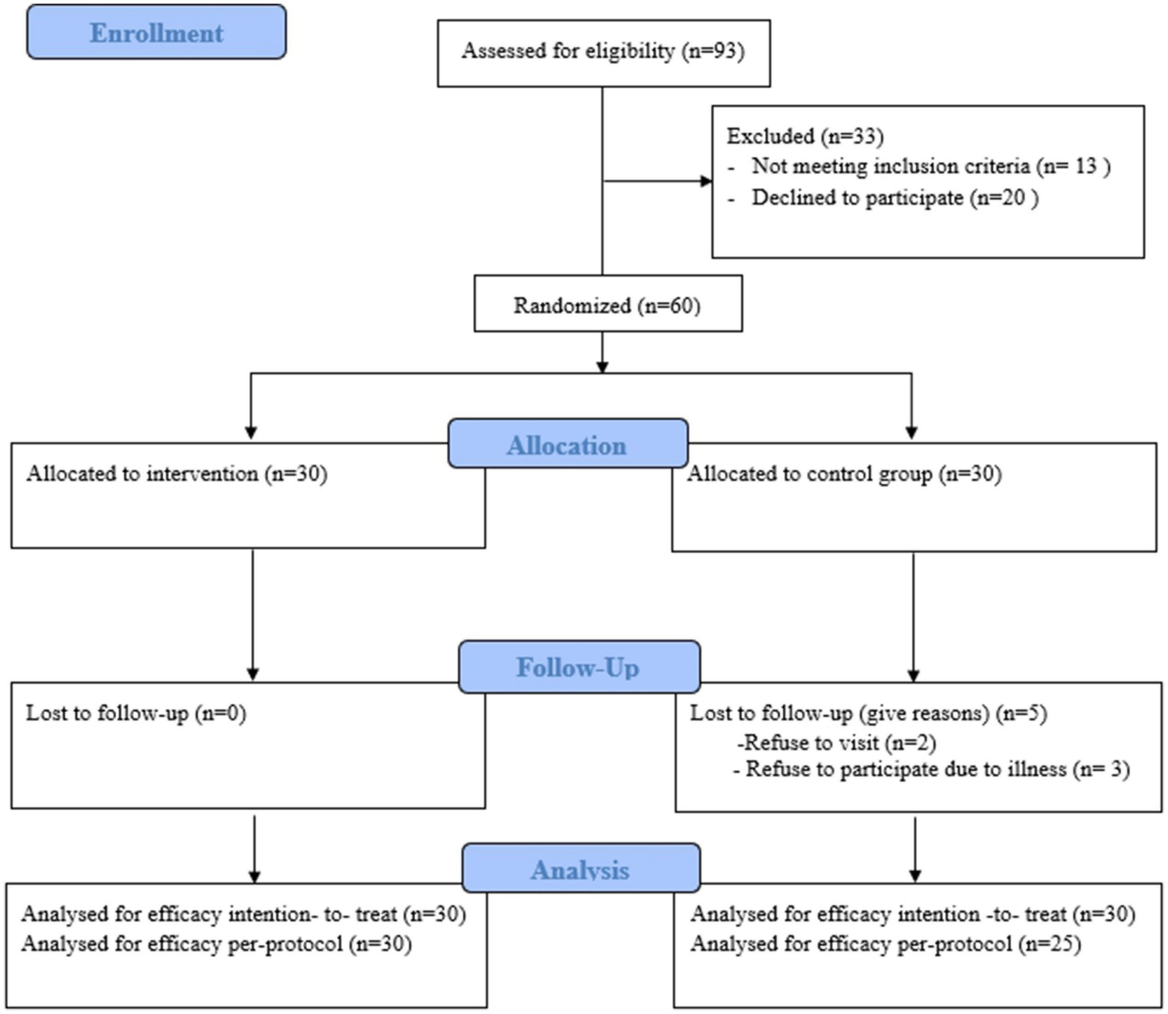
Flow diagram of the study design.

Inclusion criteria were age of 60 years or older, residing in Incheon, and being able to walk without the use of wheelchair or walker. Exclusion criteria included diagnosed major chronic diseases, walking disabilities, and cognitive impairments affecting participation in the exercise program. Among the recruited participants, 13 did not meet the inclusion criteria and 20 declined to participate. The 60 remaining participants were randomly assigned to either the exercise or control group (Figure 1). All participants were informed of their right to withdraw at any time.

### Randomizations

The participants were assigned to the experimental or control group through block randomization, which operated by randomizing within 15 blocks of size 4. The random blocks were created using an Excel number generator. Group assignments were distinguished by individual ID numbers in an Excel spreadsheet. This password-protected spreadsheet was accessible only to a third-party research assistant unaware of participant recruitment, screening, and informed consent details. For concealed allocation, a third-party assistant unrelated to the study conducted the allocation process, and allocation was only revealed to participants and therapists by phone call to the study center after the baseline measurement.

### Blinding

Assessors were blinded as follows. The initial visit for selecting potential study participants and conducting baseline measurements was in each case carried out by a community nurse responsible for assessments and an independent allocator. After the initial measurements, the assessor departed, and the allocator disclosed the allocation results after having called the study center. Participants and allocators were instructed not to disclose allocation information to assessors. To maintain blinding of assessors, all following measurements were conducted by the same community nurses affiliated with the public health center and otherwise uninvolved in the study.

### Intervention

Participants were visited in person at their homes for the installation of wireless internet and at the public health center for application usage training, during which baseline measurements were also collected. Follow-up measurements were conducted in person at the 4-week and 8-week intervals. Up to 1 week before the start of the study, participants received free wireless internet installation at home, along with necessary equipment such as tablet PCs, fitness bands, weighing scales, and blood pressure monitors.

We conducted instructional sessions to teach participants how to use the equipment via the ‘Smart Healthcare’ mobile application which was specifically developed for the ‘SHe CoFFEE’ program to enhance usability for older adults. Recognizing the value of information and communication technology through in-person training for older adults can impact their intention to use smart health technology (25), we aimed to provide accessible and user-friendly smart health applications. Using the ‘Smart Healthcare’ app, participants were instructed to perform real-time exercises and walking for fall prevention, as well as individual exercises through videos.

To support the ‘SHe CoFFEE’ program, we initially assisted older individuals in becoming familiar with smart healthcare technologies. We offered continuous support, including guidance and essential resources, to help participants comfortably use tablets and health bands for regular exercise.

The “SHe CoFFEE” program consisted of continuous feedback, self-monitoring, and fall prevention exercises (Table 2). We provided continuous feedback to enhance the adherence of older adults to the exercise and their sense of accomplishment. This feedback included: (1) assigning daily tasks and setting activity alarms, including exercise, (2) timely reminders for daily weight, blood pressure, and nutrition measurements, (3) educational nutrition and exercise videos delivered via tablet PC twice weekly, and (4) daily medication administration reminders.

**Table 2:** Contents of the SHe CoFFEE program.

Intervention contents	Description
Continuous feedback	Activity time using an alarm (5 days/week)
	Measurement time (5 days/week)
	Education time (2 days/week)
	Medication adherence (5 days/week)
Self-monitoring	Weight measurement (5 days/week)
	Blood pressure measurement (5 days/week)
	Nutrition intake (5 days/week)
Fall prevention exercise	Real-time online group exercise (3 days/week, for 30 min)
	Home-based Individual exercises:
	Walking (5 days/week, for 40 min or 7,000 steps)
	Exercise using a video (2 days/week)

A self-monitoring assessment was conducted to enhance self-motivation for health management, involving monitoring blood pressure, weight, and nutrition. Older adults were prompted to participate in activities facilitated by the smart health application through tablet alarms set at specific times based on the day of the week.

Fall prevention exercise consisted of group and individual sessions. Group exercises, aimed at enhancing muscle strength in older adults, and included neck, shoulder, full-body, and muscle-strengthening exercises, as well as walking. These sessions, lasting approximately 30 min, were conducted three times a week via real-time online video conferences by expert instructors and researchers. Group exercise sessions were accessed via the pre-installed smart healthcare app on the provided tablet, following a pre-scheduled timetable. Older adults received training on accessing these sessions by pressing preset alarms on the app. For individual exercises, we provided fitness bands to older participants, encouraging them to walk for at least 40 min daily or engage in walking activities at least five times a week. This encouragement was tailored to each person’s ability, to exceed 7,000 steps per day. Additionally, a fall intervention exercise video encouraged participation in individual exercise sessions. Participants also received a small reward for each visit to the public health center. The “SHe CoFFEE” program lasted 8 weeks, starting from July 4th to August 22nd, 2022. Aside from the in-person training for internet installation, app education, and measurements, participants could make phone calls and send text messages to a designated call center. Communication via messages on the tablet was also available. Additionally, during real-time exercise sessions, participants were able to communicate with researchers through video calls.

To support the participants’ continuing, use of ICT and to encourage ongoing physical activity, the provided tablet PCs and health bands were given to them as their own. Additionally, older adults offered free internet access for 2 years starting from the beginning of the study.

### Control group

Control group participants were on a waiting list for 8 weeks within which they did not receive any interventions. After the conclusion of the trial, they received the same intervention as the experimental group (Table 1).

### Outcome measurements

At baseline, older adults answered general and health-related questions and basic physical measurements were taken. Throughout their visits, participants underwent assessments, including the Timed Up and Go test (TUG), 10-meter walking test (10MWT), 30-s chair stand test (30CST), along with evaluations of fear of falling, balance confidence, and quality of life across the three sessions.

#### Primary outcomes

The primary outcome in this study was changes in mobility over 8 weeks as measured using TUG. The TUG test consists of the following tasks: standing up from a standard armchair, walking a distance of 3 m, turning around, and sitting back down (26). A shorter TUG time indicates that an individual can effectively transfer from a seated position, move with speed, demonstrate agility in rotational movements, and sit down with stability. TUG is a tool used to assess older adults’ gait speed and balance.

#### Secondary outcomes

##### Walking ability

Participants were assessed for walking speed using the 10-Meter Walk Test (10MWT), during which they walked a total of 14 m, and the time taken to walk 10 m excluding the first and last 2 m was measured (27).

##### Leg strength and endurance

We used 30CST to measure leg strength and endurance in older adults. 30CST involves counting the number of times the patient can come to a full standing position from a sitting position in 30 s (28).

##### Fear of falling

Fear of falling was measured with the Korean version of the Falls Efficacy Scale – International (KFES-I) (29). FES-I measures “concern” about falling, a concept connected to fear (30). A higher score implies a stronger fear of falling (31).

##### Balance confidence

Balance confidence was measured using the Activities-specific Balance Confidence Scale (ABC). On this scale, 0% indicates no confidence in balance while 100% indicates complete confidence (32).

##### Quality of life

Quality of life was measured using the Korean version of Euro Qol 5 Dimension 5 Level (EQ-5D-5L) (33). This tool evaluates five dimensions: mobility, self-care, manual activities, pain/discomfort, and anxiety/depression. A higher score indicates better health, with scores ranging from 0 to 1, where 1 represents the highest level of health.

#### Sample size

The required sample size was calculated using G power (34). To calculate the sample size, the alpha error and power were set at 0.05 and 0.95, respectively. A moderate effect size of 0.25 was set based on Cohen’s methods (35). Therefore, a total sample size of 44 participants was required. With an anticipated dropout rate of 30%, we aimed to recruit a total of 60 participants.

### Statistical analysis

Continuous data are presented using mean and SD while categorical data are presented using count (percentage). The primary outcome, TUG at 8 weeks, was analyzed using intention-to-treat (ITT) analysis. To address missing data, we assumed they were missing at random and employed Multiple Imputation by Chained Equations (MICE), using a multivariate normal chained equation on 20 sets. The mean differences (MD) were combined using Rubin’s rules to obtain a pooled effect with an associated 95% CI.

For the main analysis, we used an ANCOVA-style general linear model to regressing TUG at endpoint on group membership and baseline TUG score. To assess the robustness of our findings, sensitivity analysis adjusted for age and sex in addition. Moreover, complete case analysis on unimputed data and per-protocol analysis, which included only those participants who completed the study according to the protocol, were conducted. All statistical analyses were performed using SPSS, version 25.0 (IBM Corporation) and Two-sided *p* < 0.05 was considered statistically significant.

## Results

Of 93 participants screened, 33 were excluded. At 8 weeks, data from all randomized participants were assessed in an ITT analysis. For the complete case analysis, 30 of 30 participants (100%) in the intervention group and 25 of 30 (83%) in the control group were analyzed. Five control group members withdrew from the study: two due to COVID-19 concerns regarding public exposure or travel and three due to illness. The participant flowchart is presented in Figure 1.

The baseline demographic and clinical characteristics of participants are summarized in Table 3. The intervention group’s average age was 71.77 ± 6.58 years, with six males and 24 females. The control group’s average age was 70.83 ± 6.58 years, with seven males and 23 females.

**Table 3:** Homogeneity of characteristics between groups.

		Experimental group (*N* = 30)	Control group (*N* = 30)	*t* or *χ*^2^	*p*
		Mean ± SD or *n* (%)	Mean ± SD or *n* (%)		
Age (years)		71.77 ± 6.58	70.83 ± 6.58	0.55	0.59
Sex	Male	6 (20)	7 (23.3)	0.10	0.59
	Female	24 (80)	23 (76.7)		
Education	≤Elementary school	8 (26.7)	8 (26.7)	6.62	0.66
	Middle school	10 (33.3)	7 (23.3)		
	High school	6 (20)	10 (33.3)		
	≥College	6 (20)	5 (16.7)		
Living status	Alone	12 (40)	15 (50)	0.61	0.44
	With family	18 (60)	15 (50)		
BMI (kg/m^2^)		25.08 ± 3.64	26.38 ± 4.64	1.21	0.23
Smoke	Yes	1 (3.3)	4 (13.3)	1.96	0.16
	No	29 (96.7)	26 (87.7)		
Drink	Yes	6 (20)	5 (16.7)	0.11	0.74
	No	24 (80)	25 (83.3)		
Number of medications taken	≤ 3	25 (83.3)	27 (90)	0.58	0.45
	≥4	5 (16.7)	3 (10)		
TUG		11.02 ± 3.66	10.21 ± 2.18	1.03	0.30
10MWT		11.78 ± 3.16	11.78 ± 3.51	0.00	1.00
30CST		12.57 ± 3.91	12.40 ± 4.57	0.15	0.88
KFES-I		21.30 ± 7.38	22.20 ± 6.71	−0.49	0.62
ABC (0–100)		80.83 ± 24.36	75.33 ± 24.80	0.87	0.39
EQ-5D-5L (0–1)		0.83 ± 0.19	0.78 ± 0.18	1.08	0.28

### Primary outcome

**Figure 2:**
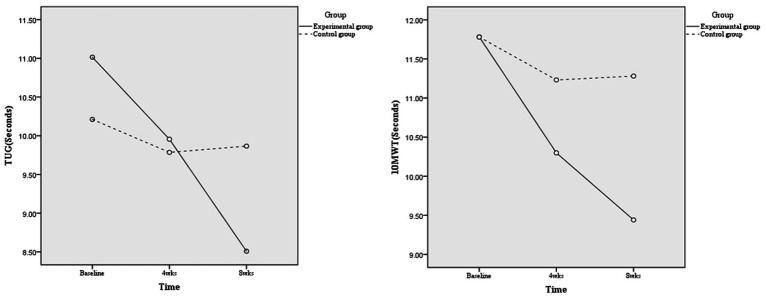
Mobility outcomes.

At the primary time point of 8 weeks, the mean difference between groups was statistically significant both in the unadjusted ITT model (mean difference, −1.48; 95% CI, −2.50 to −0.45; *p* = 0.005) and adjusted ITT model (MD, −1.87; 95% CI, −2.60 to −1.14; *p* < 0.001; *η_p_*^2^, 0.326, considered a large effect) (Table 4). In the per-protocol analysis, which included 30 participants in the intervention group and 25 in the control group, the mean difference between groups resulted in smaller but still statistically significant reductions in TUG scores in the unadjusted model (−1.12; 95% CI, −2.17 to −0.06; *p* = 0.038). The adjusted mean difference was −1.59 (95% CI, −2.35 to −0.83; p < 0.001; *η_p_*^2^, 0.262, considered a large effect), as shown in Table 4 and Figure 2.

**Table 4:** Mean difference in TUG between intervention and control groups by unadjusted and adjusted intention-to-treat analysis and per-protocol analysis.

	TUG, mean (SD)		Mean difference between groups (95% CI)			
Time	Control	Experimental	Unadjusted	*p* value	Adjusted	*p* value
Intention-to-treat analysis						
Baseline	10.21 (2.18)	11.02 (3.66)	NA	NA	NA	NA
4 weeks	9.77 (2.23)	9.96 (2.97)	0.18 (−1.18 to 1.54)	0.791	−0.37 (−1.29 to 0.55)	0.430
8 weeks	9.99 (1.89)	8.51 (2.07)	−1.48 (−2.50 to −0.45)	0.005	−1.87 (−2.60 to −1.14)	<0.001
Per-protocol analysis						
Baseline	10.05 (1.95)	11.02 (3.66)	NA	NA	NA	NA
4 weeks	9.53 (2.18)	9.96 (2.97)	0.43 (−1.01 to 1.86)	0.554	−0.25 (−1.24 to 0.74)	0.615
8 weeks	9.63 (1.76)	8.51 (2.07)	−1.12 (−2.17 to −0.06)	0.009	−1.59 (−2.35 to −0.83)	<0.001

NA: Not applicable; TUG: Timed-Up and Go test.

### Secondary outcome

At the 8 weeks, the intervention group showed a significant improvement in 10MWT, with MD of −1.88 (95% CI, −3.07 to −0.69; *p* = 0.003; *η_p_*^2^ = 0.146) in the unadjusted analysis and − 1.87 (95% CI, −2.60 to −1.14; *p* < 0.001; *η_p_*^2^ = 0.312) in the adjusted analysis. For the 30CST, the MD at 8 weeks was 4.30 (95% CI, 1.62–6.98; *p* = 0.002; *η_p_*^2^ = 0.151) unadjusted and 4.92 (95% CI, 2.44–7.40; *p* < 0.001; *η_p_*^2^ = 0.223) adjusted, indicating significant improvements. The KFES-I results at 8 weeks also showed significant improvements with MD of −3.60 (95% CI, −6.66 to −0.54; *p* = 0.022; *η_p_*^2^ = 0.088) unadjusted and − 4.20 (95% CI, −7.12 to −1.29; *p* = 0.005; *η_p_*^2^ = 0.132) adjusted. ABC scores were significantly better at 8 weeks in both unadjusted (MD, 11.90; 95% CI, 0.36–23.44; *p* = 0.042; *η_p_*^2^ = 0.069) and adjusted (MD,13.95; 95% CI, 2.74–25.16; *p* = 0.016; *η_p_*^2^ = 0.102) analyses. EQ-5D-5L did not show significant changes at 8 weeks with MD of 0.05 (95% CI, −0.03 to 0.14; *p* = 0.258) unadjusted and 0.07 (95% CI, −0.01 to 0.15; *p* = 0.096) adjusted (Table 5). In the per-protocol analysis, we observed statistically significant improvements in 10MWT and 30CST at 8 weeks. Specifically, the 10 MWT showed a significant reduction in time with an MD of −1.42 (95% CI, −2.60 to −0.024; *p* = 0.019; *η_p_*^2^ = 0.140) in the unadjusted model and the adjusted MD was −1.91 (95% CI, −2.84 to −0.98; *p* < 0.001; *η_p_*^2^ = 0.292). Similarly, the 30CST demonstrated notable improvements, with an MD of 3.41 (95% CI, 0.62–6.21; *p* = 0.018; *η_p_*^2^ = 0.131) unadjusted and 4.15 (95% CI, 1.52–6.78; *p* = 0.003; *η_p_*^2^ = 0.199) adjusted. In contrast, the KFES-I and ABC scales did not show significant differences between groups in the per-protocol analysis. The KFES-I’s MD was −1.39 (95% CI, −3.84 to 1.06, *p* = 0.259) unadjusted and −1.96 (95% CI, −4.31 to 0.39, *p* = 0.100) adjusted, while the ABC scale had an MD of 4.00 (95% CI, −6.05 to 14.04, *p* = 0.429) unadjusted and 6.46 (95% CI, −3.05 to 16.00, *p* = 0.179) adjusted. Furthermore, the EQ-5D-5L results at 8 weeks showed no significant changes with an MD of −0.01 (95% CI, −0.09 to 0.08; *p* = 0.900) unadjusted and 0.01 (95% CI, −0.06 to 0.08; *p* = 0.707) adjusted (Supplementary Table 1).

**Table 5:** Mean difference of secondary outcomes between intervention and control groups by unadjusted and adjusted intention-to-treat analysis.

	Mean (SD)		Mean difference between groups (95% CI)			
Time	Control	Experimental	Unadjusted	*p* value	Adjusted	*p* value
10MWT						
4 weeks	11.26 (2.81)	10.30 (2.64)	−0.97 (−2.37 to 0.44)	0.175	−0.37 (−1.28 to 0.55)	0.430
8 weeks	11.32 (2.49)	9.44 (2.12)	−1.88 (−3.07 to −0.69)	0.003	−1.87 (−2.60 to −1.14)	<0.001
30CST						
4 weeks	12.96 (3.67)	15.17 (5.04)	2.20 (−0.07 to 4.48)	0.058	2.84 (0.85 to 4.84)	0.006
8 weeks	12.39 (4.96)	16.70 (5.40)	4.30 (1.62 to 6.98)	0.002	4.92 (2.44 to 7.40)	<0.001
KFES-I						
4 weeks	23.43 (8.91)	20.03 (5.92)	−3.40 (−7.31 to 0.51)	0.087	−4.37 (−7.86 to −0.87)	0.015
8 weeks	21.77 (7.44)	18.17 (3.72)	−3.60 (−6.66 to −0.54)	0.022	−4.20 (−7.12 to −1.29)	0.005
ABC						
4 weeks	82.46 (22.19)	86.92 (18.49)	4.46 (−6.10 to 15.02)	0.402	6.60 (−3.38 to 16.57)	0.191
8 weeks	78.13 (27.19)	90.02 (15.72)	11.90 (0.36 to 23.44)	0.044	13.95 (2.74 to 25.16)	0.016
EQ-5D-5L						
4 weeks	0.81 (0.18)	0.83 (0.16)	0.03 (−0.06 to 0.11)	0.569	0.04 (−0.04 to 0.13)	0.320
8 weeks	0.81 (0.18)	0.87 (0.16)	0.05 (−0.03 to 0.14)	0.258	0.07 (−0.01 to 0.15)	0.096

10MWT: 10-Meter Walk Test; 30CST: 30-Second Chair Stand Test; ABC: Activities-specific Balance Confidence Scale; EQ-5D-5L: EuroQol 5-Dimension 5-Level; KFES-I: Korean version of the Falls Efficacy Scale-International.

## Discussion

The SHe CoFFEE program in this study effectively enhanced mobility among older adults residing in local communities who are not familiar with online technology. Conducted over 8 weeks, this program resulted in notable improvements in the primary outcome, TUG. Additionally, significant results were observed for the secondary outcomes 10MWT and 30SCT, KFES-1, and ABC but not for EQ-5D-5L. Consistent with the present study, previous studies reported that an 8-week online home-based exercise program for older adults showed significant improvements in TUG (36) and a web-based multi-domain lifestyle intervention for older adults improved balance (37). In an online exercise intervention program conducted over 8 weeks for chronic stroke survivors, significant results were observed in the 10MWT (38). Additionally, in a study targeting older adults with mild cognitive impairment for online fall prevention exercises, the effect of the online intervention was shown for 30SCT (39).

The SHe CoFFEE program consisted of continuous feedback, self-monitoring, and fall prevention exercises using online technology. Continuous feedback which is one of the SHe CoFFEE program contents was provided to participants using messages and alarms in this study. Since frequent notifications can increase exposure to intervention content without discouraging participation (40), it can effectively encourage older adults to stay engaged. The effectiveness of the intervention and the creation of positive emotions are crucial for the compliance of older adults with the intervention (41). Therefore, continuous feedback appears to have a positive impact on intervention participation especially when conducting interventions with older adults in this study.

Self-monitoring is a crucial element of health promotion and self-monitoring with online technology has the potential to promote health in an individual’s daily life (42). Consistent with this study, the previous study about wireless self-monitoring programs for hypertension patients insisted that self-monitoring was associated with improvements in blood pressure control (43). Also, in line with this study, individuals classified as obese experienced weight loss by using self-monitoring to record body weight, step count, and diet (44). Participants of this study were provided with tablet PCs, weighing scales, and blood pressure monitors and were trained on how to use the equipment to conduct a self-monitoring assessment such as blood pressure and weight. Online technology such as tablet PCs seems to provide treatment for psychosocial and health behaviors and giving a new technological device and education on how to use might increase excitement and adherence (42). In addition, self-monitoring can motivate participation in long-term health programs (45). We observed that none of the intervention group dropped out and participants felt more comfortable with online interventions while maintaining their health. Therefore, self-monitoring with online technology can be an important factor in encouraging older adults to participate in long-term health programs.

The fall prevention exercise in the SHe CoFFEE program consisted of real-time online group exercise and home-based individual exercise. Real-time online group exercise improves physical function and allows for individualized adjustment of exercise levels (46). It also encourages social interaction, particularly important for those without a spouse or living alone (47). In our study during the COVID-19 pandemic, we promoted exercise participation through group exercise, fostering social interaction. Social interaction is a key to enhancing exercise adherence (48, 49). In this study, the experimental group’s full participation in the program for the entire 8 weeks was possibly positively influenced by the group elements of the online exercise.

In our study, KFES-I and ABC showed inconsistent effects of the program according to intention-to-treat analysis and per-protocol analysis. In a study involving older adults who underwent 10 weeks of home balance training, improvements were observed in ABC scores and gait speed (50). However, in an online exercise intervention targeting older adult women, fall efficacy did not show a significant difference (51). Consistent results were not obtained in our analysis as well, indicating the need for further research. Additionally, in our results, the per-protocol analysis showed that KFES-I and ABC were not statistically significant, unlike in the intention-to-treat analysis. Furthermore, typically in per-protocol analysis, there should be a greater difference compared to intention-to-treat analysis. However, in our case, a larger difference was observed in the intention-to-treat analysis. This is speculated to be due to the exclusion of 5 subjects from the control group, resulting in a less pronounced difference when they were not included. It is hypothesized that the reasons for their exclusion might be related to illness or reluctance to go out.

Regarding the quality of life, no significant differences were found between the experimental and control groups. A previous study that implemented multidomain interventions for 6 months (37) also did not find differences in quality of life between the intervention and control groups. Various factors, such as health, independence, finances, social connections, healthcare, housing, community resources, and decision-making, impact the quality of life for older adults (52). Accordingly, likely, our SHe CoFFEE program did not show a statistically significant impact on overall quality of life because of various external factors. Controlling for various other factors aside from the SHe CoFFEE program or extending the study duration to 12 months (53) might have revealed significant effects on quality of life.

### Limitations and strengths

This study is a pilot study with a small number of participants, and data was collected from a single local community, which may potentially impact the generalizability of the findings. Additionally, using a block size of 4 for random allocation might introduce bias during eligibility checks. Despite conducting our interventions online, older adults expressed concerns about brief meetings for measurements due to the ongoing COVID-19 situation, making it challenging to secure a sufficient sample size for subgroup analysis. Moreover, we could not conduct more frequent TUG assessments due to COVID-19 constraints. The gender distribution in the participant group was also unbalanced, with a significantly higher proportion of women. Future studies should aim for a more balanced gender ratio and include a larger population for subgroup analysis.

Due to the severity of the pandemic period, there were missing data during the follow-up, which poses limitations. Furthermore, because of the absence of studies on MCID specific to Korea, we relied on clinical populations from India as a basis, for determining the MCID for TUG. We cannot ascertain that this difference is really meaningful in the Korean population. Additionally, younger family members could potentially help older adults adapt to ICT. It would be beneficial to explore the impact of family generational composition in future research.

These limitations are balanced by several strengths. One of the strengths of our study is the development of a user-friendly mobile application. We also provided continuous education and support to older participants unfamiliar with the internet and devices. Additionally, we encouraged older adults to monitor their health themselves, serving as the starting point for ongoing health management. Furthermore, we offered exercise programs for older adults, enabling them to maintain physical activity during the pandemic period.

## Conclusion

In this study, an 8-week “SHe CoFFEE” program targeting older adults was found to have a positive impact on the mobility of older individuals in the community. The use of continuous feedback and a user-friendly application in the “SHe CoFFEE” program appears to be a feasible intervention for improving the mobility of older adults. However, longer intervention periods and larger sample sizes seem necessary to demonstrate clinically significant results.

## Data Availability

The original contributions presented in the study are included in the article/Supplementary material, further inquiries can be directed to the corresponding authors.
