# Supplementary material for: The effect of the Smart Health Continuous Feedback For Elderly Exercise (SHe CoFFEE) program on mobility: a randomized controlled pilot study
Source: Front Public Health. 2024 Sep 13;12:1442064. doi: 10.3389/fpubh.2024.1442064 (PMC11427408; doi:10.3389/fpubh.2024.1442064)
Supplement: Supplementary file 1 [file Table_1.docx]

S1. Mean Difference of secondary outcomes between intervention and control groups by unadjusted and adjusted per-protocol analysis

|  | Mean (SD) | |  | Mean difference between groups (95% CI) | | | |
| --- | --- | --- | --- | --- | --- | --- | --- |
| Time | Control | Experimental |  | Unadjusted | P value | Adjusted | P value |
| **10MWT** |  |  |  |  |  |  |  |
| 4 weeks | 10.84 (2.71) | 10.30 (2.64) |  | -0.54 (-1.99 to 0.91) | .457 | -1.10 (-2.30 to 0.09) | .069 |
| 8 weeks | 10.86 (2.24) | 9.44 (2.12) |  | -1.42 (-2.60 to -0.24) | .019 | -1.91 (-2.84 to -0.98) | < .001 |
| **30SCT** |  |  |  |  |  |  |  |
| 4 weeks | 13.52 (3.50) | 15.17 (5.04) |  | 1.65 (-0.75 to 4.04) | .174 | 2.37 (0.21 to 4.54) | .032 |
| 8 weeks | 13.28 (4.83) | 16.70 (5.40) |  | 3.41 (0.62 to 6.21) | .018 | 4.15 (1.52 to 6.78) | .003 |
| **FES** |  |  |  |  |  |  |  |
| 4 weeks | 21.52 (7.67) | 20.03 (5.92) |  | -1.49 (-5.17 to 2.19) | .421 | -2.46 (-5.81 to 0.90) | .147 |
| 8 weeks | 19.56 (5.31) | 18.17 (3.72) |  | -1.39 (-3.84 to 1.06) | .259 | -1.96 (-4.31 to 0.39) | .100 |
| **ABC** |  |  |  |  |  |  |  |
| 4 weeks | 87.68 (18.17) | 86.92 (18.49) |  | -0.76 (-10.72 to 9.21) | .879 | 1.23 (-8.45 to 10.90) | .800 |
| 8 weeks | 86.03 (21.37) | 90.02 (15.72) |  | 4.00 (-6.05 to 14.04) | .429 | 6.46 (-3.05 to 16.00) | .179 |
| **EQ-5D-5L** |  |  |  |  |  |  |  |
| 4 weeks | 0.84 (.017) | 0.83 (0.16) |  | -0.01 (-0.10 to 0.08) | .799 | 0.01 (-0.08 to 0.09) | .910 |
| 8 weeks | 0.87 (0.14) | 0.87 (0.16) |  | -0.01 (-0.09 to 0.08) | .900 | .001 (-0.06 to 0.08) | .707 |

10MWT: 10-Meter Walk Test; 30CST: 30-Second Chair Stand Test; ABC: Activities-specific Balance Confidence Scale; EQ-5D-5L: EuroQol 5-Dimension 5-Level; KFES-I: Korean version of the Falls Efficacy Scale-International.
